# Supplementary material for: Distinct DNA methylation profiles in subtypes of orofacial cleft
Source: Clin Epigenetics. 2017 Jun 8;9:63. doi: 10.1186/s13148-017-0362-2 (PMC5465456; doi:10.1186/s13148-017-0362-2)
Supplement: Supplementary file 1 — Supplementary methods and results. (DOCX 119 kb) [file 13148_2017_362_MOESM1_ESM.docx]

## Additional File 1 [Supplementary methods and results]

#### Calculating epigenetic age and age acceleration

The child’s age at biological sample collection was calculated from the child’s date of birth and date of surgery. We also predicted child’s ‘epigenetic age’ using the method developed by Horvath^[[1]](#footnote-1)^, which uses raw (not normalised) methylation values at 353 CpG sites on the Illumina HM450 array to estimate age. We uploaded raw methylation and actual age data to the online epigenetic clock calculator (http://labs.genetics.ucla.edu/horvath/dnamage/), which returned an output file containing the methylation predicted epigenetic age of each participant, estimated cell type proportions in each sample and an estimate of age acceleration. This is calculated as the residuals from a linear regression of epigenetic age on actual age at sample collection, with adjustment for estimated cell type proportions. A positive value corresponds to an individual whose epigenetic age is ahead of their actual age, and vice-versa.

#### Assessing the efficacy of a methylation-predicted prenatal tobacco exposure score

The efficacy of the methylation-predicted prenatal tobacco exposure score was assessed by running an EWAS of blood methylation regressed on the score and looking up the top CpG sites with the smallest P-values in the results of a previous maternal smoking EWAS meta-analysis^[[2]](#footnote-2)^.

The EWAS of the methylation-predicted smoking score identified several CpG sites that have previously been associated with maternal smoking in pregnancy (top ten with smallest P-values shown in Table 1), so we can be reasonably confident that the smoking score provides an objective measure of the child’s (prenatal) tobacco exposure. Conversely, the top ten CpGs with the smallest P-values from an EWAS of self-reported maternal smoking around the time of conception did not contain any CpGs that have previously been associated with tobacco smoke exposure (Table 2).

Additional adjustment for methylation-predicted tobacco exposure score did not substantially change results of our OFC EWA studies: the Spearman correlation coefficient between regression coefficients in the main model and smoking-score-adjusted model ranged 0.98 to 0.99, with a median change in coefficients ranging from 6 to 16%.

**Table 1. Top ten CpGs with the smallest P-values in an EWAS of methylation-predicted prenatal tobacco exposure score**

| CpG | BETA | SE | P | Gene | Previously associated with maternal smoking (direction of effect) |
| --- | --- | --- | --- | --- | --- |
| cg25949550 | -0.03933 | 0.001668 | 1.05E-49 | *CNTNAP2* | TRUE (-) |
| cg05575921 | -0.04488 | 0.007318 | 8.94E-09 | *AHRR* | TRUE (-) |
| cg17835016 | 0.052397 | 0.009803 | 3.75E-07 | *F10* | FALSE |
| cg04180046 | 0.101677 | 0.019729 | 8.87E-07 | *MYO1G* | TRUE (+) |
| cg15742848 | -0.04682 | 0.0093 | 1.51E-06 |  | FALSE |
| cg05799169 | -0.03802 | 0.007673 | 2.13E-06 | *PLXNB2* | FALSE |
| cg00794227 | 0.009965 | 0.002027 | 2.52E-06 | *PHACTR4* | FALSE |
| cg18613421 | 0.033871 | 0.007002 | 3.53E-06 | *WDR72* | FALSE |
| cg02936263 | 0.033611 | 0.00718 | 6.85E-06 | *CCL7* | FALSE |
| cg22361075 | -0.02094 | 0.004497 | 7.63E-06 | *GPSM2* | FALSE |

**Table 2. Top ten CpGs with the smallest P-values in an EWAS of self-reported maternal smoking around the time of conception**

| CpG | BETA | SE | P | Gene | Previously associated with maternal smoking (direction of effect) |
| --- | --- | --- | --- | --- | --- |
| ch.15.75593923F | 0.020795 | 0.003081 | 9.30E-08 |  | FALSE |
| cg23871860 | -0.06596 | 0.010927 | 7.72E-07 | *FBXL3* | FALSE |
| cg06733329 | 0.011267 | 0.001925 | 1.34E-06 | *MXD3* | FALSE |
| cg10635145 | 0.109274 | 0.019942 | 4.09E-06 | *BDNF* | FALSE |
| cg06490869 | 0.016523 | 0.003008 | 4.31E-06 | *CRYM* | FALSE |
| cg26963545 | -0.0091 | 0.001685 | 5.20E-06 | *NEURL4;GPS2* | FALSE |
| cg10861135 | 0.187296 | 0.034772 | 5.41E-06 |  | FALSE |
| cg04675861 | 0.004015 | 0.000747 | 5.59E-06 |  | FALSE |
| cg04158367 | 0.008841 | 0.00162 | 8.97E-06 | *GFI1;GFI1* | FALSE |
| cg26697583 | 0.141123 | 0.0278 | 1.37E-05 |  | FALSE |

#### Sensitivity analysis: a different approach to adjust for batch and cell types

Results from the main EWAS models, where technical variables and cellular heterogeneity were corrected for using surrogate variables, agreed reasonably well with results from a model adjusting for chip number and cell counts estimated using the Houseman method (Spearman correlation coefficients ranging 0.50 to 0.78, median change in coefficients ranging 65 to 105%).

#### Assessing whether ‘age-related CpGs’ are associated with age, independent of OFC subtype

Children with CLO and CLP were younger than children with CPO, so we considered that some of our OFC-associated CpGs might be better explained by differences in age than differences in OFC subtype. To remove the influence of age, we first carried out an EWAS of age at sampling, using all the participants in our sample. In this EWAS, child’s age in months was modelled as the exposure with methylation as the outcome. The model was adjusted for 10 surrogate variables for technical batch and cellular heterogeneity. We then ‘filtered out’ any age-related CpGs (29,984 CpGs with P<0.05 in the age-at-sampling EWAS) by setting their P-values for the OFC EWASs to 1.

In order to provide more confidence that these ‘age-related’ CpGs really are associated with age (rather than OFC subtype), we ran three separate EWASs of age at sampling in each OFC subgroup and meta-analysed the results using METAL^[[3]](#footnote-3)^. At the 29,984 age-associated CpGs, there was little evidence of heterogeneity between OFC groups: At 63% of these CpGs the direction of association with age was the same in all three groups and only 6% of CpGs had a P-value for heterogeneity <0.05. This suggests that these CpGs are truly associated with age. Further support for this is provided by the observation that many of the top ‘age-related’ CpGs have previously been reported as differentially methylated in association with age in infancy^[[4]](#footnote-4)^.

*Attempting to carry out a case-control analysis using publicly-available data*

The Cleft Collective is a case-only cohort, so data on controls were not collected. Ideally, we would have liked to compare DNA methylation in controls compared to cases, so we explored options to identify an appropriate source of controls. Biological samples available to us included cord blood samples from babies in the Avon Longitudinal Study of Parents and Children (ALSPAC) and from their offspring in the second generation of the study (ALSPAC-G2). However, we considered that the differences in age and tissue type between individuals in ALSPAC/ALSPAC-G2 and the Cleft Collective would be too difficult to disentangle from any association with OFC subtype.

We also explored options to use publicly-available Illumnia 450k methylation data from children without a cleft. We searched Gene Expression Omnibus to find studies with the appropriate data on blood samples from children around the same age as the children in the Cleft Collective (around 3 to 20 months). We identified two studies for which the raw data were available: accession numbers GSE62219^4^ and GSE67444^5^. There was still potential confounding by technical batch, population, study protocol and tissue (the Cleft Collective and GSE62219 used peripheral blood leukocytes, GSE67444 used dried blood spots). However, we downloaded the data and applied our pre-processing pipeline as described in the manuscript. We normalised both public datasets together with our own from the Cleft Collective using Functional Normalisation. This increased the overlap between the three datasets in Principal Component (PC) plots, but there were still clear clusters by study and PCs were strongly associated with study (Figure 1).


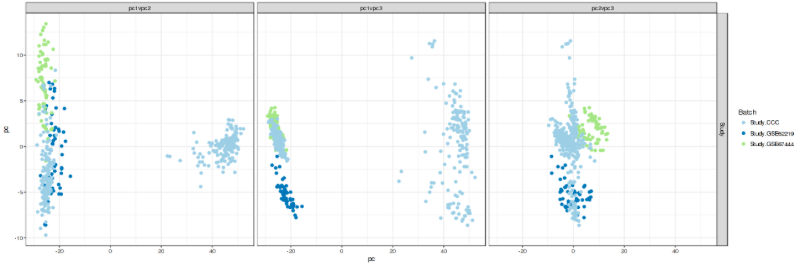


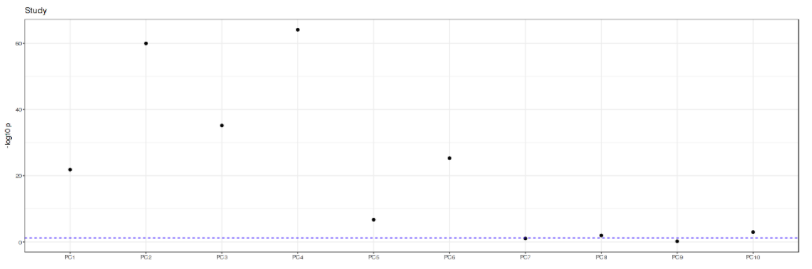


**Figure 1. Principal components are strongly associated with study. The distinct clusters within the Cleft Collective cohort (pale blue) are due to differences in lip/palate vs blood.**

Nevertheless, we attempted to run an EWAS comparing blood data from cases and controls. The EWAS was carried out as described in the manuscript, but instead of adjusting for 10 surrogate variables, we adjusted for 20 to try to capture the increased variation due to batch and study effects. The model was also adjusted for sex.

There were 268,456 CpGs with a Bonferroni-adjusted P-value<0.05 (the genomic inflation lambda was 86.8). As in the main analysis, we ran an EWAS of age and filtered out 221,997 age-related CpGs. This reduced the number of Bonferroni-significant CpGs in the case-control EWAS to 129,424. We felt that this was an unacceptable level of inflation and therefore did not consider the results of this analysis further.

1. *Horvath, S., 2013. DNA methylation age of human tissues and cell types. Genome biology, 14(10), p.R115.* [↑](#footnote-ref-1)
2. *Joubert, B.R. et al., 2016. DNA Methylation in Newborns and Maternal Smoking in Pregnancy: Genome-wide Consortium Meta-analysis. American journal of human genetics* [↑](#footnote-ref-2)
3. *Willer, C.J., Li, Y. & Abecasis, G.R., 2010. METAL: fast and efficient meta-analysis of genomewide association scans. Bioinformatics (Oxford, England), 26(17), pp.2190–1.* [↑](#footnote-ref-3)
4. *Acevedo, N. et al., 2015. Age-associated DNA methylation changes in immune genes, histone modifiers and chromatin remodeling factors within 5 years after birth in human blood leukocytes. Clinical epigenetics, 7(1), p.34.*

   *^5^ Sen A, Heredia N, Senut M-C, et al. Multigenerational epigenetic inheritance in humans: DNA methylation changes associated with maternal exposure to lead can be transmitted to the grandchildren. Sci. Rep. 5(January), 14466 (2015).* [↑](#footnote-ref-4)
